# Les teignes du cuir chevelu: étude épidémiologique dans la région de Tunis de 2012 à 2020

**DOI:** 10.11604/pamj.2022.41.168.29473

**Published:** 2022-03-01

**Authors:** Latifa Mtibaa, Faten Rabhi, Achraf Abderrahim, Nawel Baccouchi, Kahena Jaber, Hajer Fares, Abderraouf Dhaoui, Boutheina Jemli

**Affiliations:** 1 Laboratoire de Parasitologie-Mycologie, Hôpital Militaire Principal d’Instruction de Tunis, 1008 Monfleury, Tunisie,; 2 Service de Dermatologie, Hôpital Militaire Principal d´Instruction de Tunis, 1008 Monfleury, Tunisie

**Keywords:** Teigne du cuir chevelu, épidémiologie, Tunis, Tinea capitis, epidemiology, Tunis

## Abstract

Les teignes du cuir chevelu (TCC) constituent la principale mycose superficielle de l´enfant avant la puberté. La distribution des dermatophytes en cause change avec le temps et varie d´un pays à l´autre. Le but de ce travail était d´étudier les caractéristiques épidémiologiques et mycologiques des TCC dans la région de Tunis. Notre étude est rétrospective ayant porté sur 474 patients adressés pour prélèvement mycologique du cuir chevelu entre janvier 2012 et décembre 2020. Pour chaque patient, une fiche de renseignement clinique a été remplie et un prélèvement mycologique a été réalisé. L´identification des dermatophytes isolés a reposé sur des critères macroscopiques et microscopiques des colonies. L´identification moléculaire a été réalisée pour 4 isolats par Réaction en chaîne par polymérase (PCR) en temps réel en utilisant le kit DermaGenius®2.0. Nous avons colligé 210 prélèvements positifs soit une prévalence de 44,3% (n=210). Les patients de sexe masculin sont plus fréquemment atteints par les TCC (81%, n=170). L´âge moyen des patients est de 6,2±3,4 ans. Les teignes tondantes à grande plaque étaient prédominantes (88%, n=184). La sensibilité de l´examen direct était de 87% (n=182). Il s´agissait d´un parasitisme ectothrix microsporique dans (79%, n=166) des cas et endothrix trichophytique dans (7%, n=14) des cas. La culture était positive dans (98%, n=207) des cas et isolait cinq espèces de dermatophytes: Microsporum canis (87%, n=182), Trichophyton violaceum (9%, n=19). Trichophyton mentagrophytes var mentagrophytes (3%, n=6), Microsporum gypseum (0,5%, n=1) et Trichophyton verrucosum (0,5%, n=1). Notre étude montre l´émergence des dermatophytes zoophiles particulièrement M. canis. L´examen mycologique est essentiel pour la confirmation du diagnostic, la surveillance épidémiologique des dermatophytes selon les régions et la prise en charge thérapeutique.

## Abstract (en)

Tinea capitis (TC) is the most common superficial mycosis seen in children before puberty. The distribution of these dermatophytes changes over time and varies from country to country. The purpose of this study was to investigate the epidemiological and mycological characteristics of TC in Tunis. We conducted a retrospective study, involving 474 patients referred for mycological analysis of the scalp between January 2012 and December 2020. For each patient, a Clinical Information Sheet was completed, and a mycological sample was taken. The identification of isolated dermatophytes was based on macroscopic and microscopic colony criteria. Molecular real-time Polymerase Chain Reaction (PCR) detection of 4 isolates was performed using the DermaGenius®2.0 kit. We collected 210 positive samples, reflecting a prevalence of 44.3% (n=210). Male patients were more frequently affected by TC (81%, n=170). The average age of patients was 6,2±3,4 years. Ring-shaped large patches were predominant (88%, n=184). Direct examination had a sensitivity of 87% (n=182). Microsporic ectothrix parasitism was detected in (79%, n=166) of cases and trichophytic endothrix in (7%, n=14) of cases. Culture was positive in (98%, n=207) of cases and five dermatophyte species were isolated: Microsporum canis (87%, n=182), Trichophyton violaceum (9%, n=19), Trichophyton mentagrophytes var mentagrophytes (3%, n=6), Microsporum gypsum (0.5%, n=1) and Trichophyton verrucosum (0.5%, n=1). This study reveals the occurrence of zoophilic dermatophytes, in particular M. canis. Mycological examination is essential to confirm the diagnosis, conduct epidemiological surveillance of dermatophytes in the districts and for therapeutic management.

## Introduction

Les teignes du cuir chevelu (TCC) sont des infections fongiques dues à des dematophytes qui colonisent et infectent la couche superficielle du cuir chevelu. Ces dermatophytoses constituent la principale mycose superficielle de l´enfant avant la puberté avec des fréquences assez élevées allant jusqu´à 59% [1]. Leur prévalence a diminué dans les pays développés, cependant elle demeure élevée et pose un problème sérieux de santé publique dans les pays en voie de développement [2]. Etant donné que la distribution des dermatophytes en cause change avec le temps et varie d´un pays à l´autre et d´une région à l´autre au sein du même pays, nous nous proposons d´étudier les caractéristiques épidémiologiques et mycologiques des TCC dans la région de Tunis.

## Méthodes

**Type et cadre de l´étude:** il s´agit d´une étude rétrospective réalisée au laboratoire de parasitologie-mycologie de l´hôpital militaire principal d´instruction de Tunis sur une période allant du 1^er^ janvier 2012 au 31 décembre 2020.

**Population de l´étude:** nous avons inclus les patients tout âge confondu, adressés pour prélèvement mycologique du cuir chevelu pendant la période d´étude. Nous avons exclu ceux qui ont été mis sous traitement antifongique.

**Collecte de données:** pour chaque patient les données cliniques précisant l´âge, le sexe, l´aspect clinique de la lésion du cuir chevelu et la notion de contact ont été recueillies à partir des registres du laboratoire. Les données des résultats biologiques sont collectées des registres et du système informatique SYSLAB.

**Analyse du laboratoire:** les prélèvements sont réalisés à l´aide d´une pince à épiler pour récupérer les cheveux parasités, d´une curette pour racler les squames à l´intérieur des plaques. Un écouvillon est utilisé en cas de lésions inflammatoires ou séreuses. Pour chaque prélèvement, ont été réalisés de façon systématique un examen direct (ED) à la potasse à 30% et une culture sur milieu Sabouraud avec et sans actidione. Les cultures sont incubées à 27°C et examinées de façon hebdomadaire pendant 4 semaines. L´identification des dermatophytes isolés a reposé sur des critères macroscopiques et microscopiques des colonies avec repiquage sur eau gélosée. Le diagnostic mycologique a été considéré positif lorsque l´examen direct et/ou la culture étaient positifs. Nous avons procédé à une identification moléculaire pour une souche de *M. canis*, une souche de *T. mentgrophytes*, une souche de *T. violaceum* et une souche de *T. verrucosum* par PCR en temps réel en utilisant le kit DermaGenius® 2.0.

**Analyse statistique:** les données épidémiologiques ont été saisies sur le logiciel Microsoft Excel 2010 et analysées par le logiciel SPSS version 22.0. Nous avons calculé des fréquences simples et des fréquences relatives (pourcentages) pour les variables qualitatives. La mesure de l´accord entre deux caractères qualitatifs a été réalisée par l´indice de concordance Kappa. Nous avons calculé des moyennes, des médianes et des écarts types et déterminé les valeurs extrêmes (minimum et maximum) des variables quantitatives.

**Considérations éthiques:** les règles mondiales globales relatives au respect de la confidentialité et à la protection des données spécifiques aux patients ont été prises en compte lors de ce travail. Nous avons obtenu le consentement éclairé des patients et l'autorisation du comité d'Ethique de l'Hôpital Militaire de Tunis.

## Résultats

### Caractéristiques sociodémographiques

**Figure 1:**
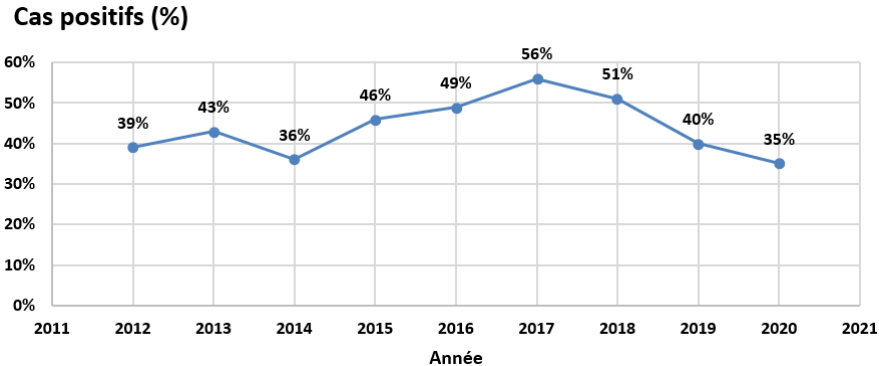
incidence annuelle des teignes du cuir chevelu entre 2012 et 2020

**Figure 2:**
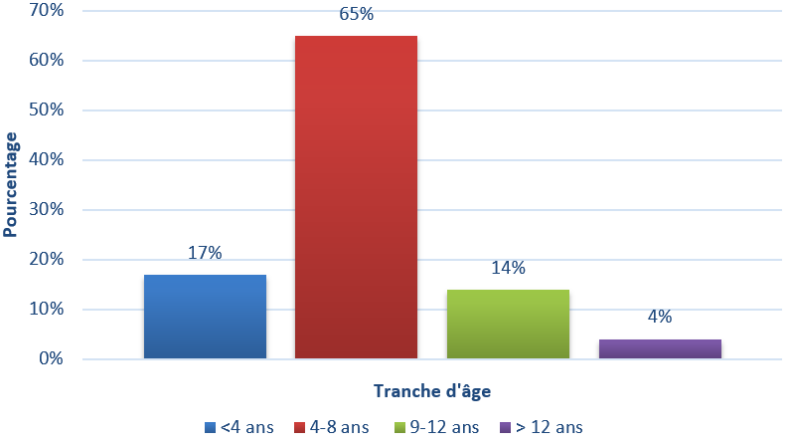
répartition des teignes du cuir chevelu selon la tranche d’âge

Pendant la période d´étude, nous avons reçu 474 malades adressés pour suspicion de TCC. Le diagnostic a été confirmé par l´examen mycologique dans 44,3% des cas (n=210).L´incidence annuelle a connu son pic en 2017 (56%, n=117) et son taux le plus bas en 2020 (35%, n=73) (Figure 1). Les patients de sexe masculin sont beaucoup plus impliqués par les teignes (81%, n=170) que le sexe féminin (19%, n=39). L´âge moyen des patients est de 6,2±3,4 ans avec des extrêmes de 1 à 29 ans. La tranche d´âge la plus touchée est celle située entre 4 et 8 ans (65%, n= 136). A partir de l´âge 9 ans, les cas de TCC diminuent (18%, n=37) (Figure 2).

### Caractéristiques cliniques

Les teignes tondantes à grandes plaques étaient prédominantes dans 88% (n=184) des cas tandis que les teignes tondantes à petites plaques et inflammatoires représentaient 9% (n=18) et 3% (n=6) respectivement. Le contact avec les animaux de compagnie a été retrouvé chez 37% (n=77) des cas. Ces animaux sont essentiellement des chats et des chiens ayant un contact étroit avec les patients.

### Caractéristiques mycologiques

La sensibilité de l´examen direct était de 87% (n=182). Il s´agissait d´un parasitisme ectothrix microsporique dans 79% des cas (n=166) et endothrix trichophytique dans 7% (n=14) des cas (Tableau 1). La concordance entre l´aspect clinique de la teigne et l´examen direct était bonne (indice kappa=0.8) (Tableau 2). La culture était positive dans 98% (n=205) des cas avec une bonne concordance entre le résultat de l´examen direct et de la culture (indice kappa=0.84) (Tableau 1). Cinq espèces de dermatophytes ont été isolées: *Microsporum canis, Trichophyton violaceum, Trichophyton* mentagrophytes var mentagrophytes, *Microsporum gypseum* et *Trichophyton verrucosum. M. canis* était prédominante dans 87% (n=182) des cas (Tableau 3). La PCR en temps réel a confirmé l´espèce identifiée dans 100% des cas (n=4).

**Tableau 1:** résultats de l´examen direct et de la culture

		Culture		
		Négative	Positive	Total
**Examen direct**	Négatif	264	28	292
	Positif	3	179	182
	Total	267	207	474

**Tableau 2:** concordance entre l´aspect clinique de la teigne et le résultat de l’examen direct

Aspect clinique/ Résultat examen direct	Teigne tondante à grande plaque	Teigne tondante à petite plaque	Teigne inflammatoire	Total
	Effectif (%)	Effectif (%)	Effectif (%)	
Négatif	23 (12%)	3 (16%)	2(29%)	28
Parasitisme endothrix	0	16 (84%)	0	16
Parasitisme ectothrix	161 (88%)	0	5 (71%)	166
Total	184 (100%)	19(100%)	7(100%)	**210**

**Tableau 3:** dermatophytes isolés de la culture

Espèce de dermatophyte	Nombre de cas	Pourcentage
***M. canis***	181	87%
***T. violaceum***	19	9%
***T. mentagrophytes var mentagrophytes***	5	3%
***M. gypseum***	1	0,5%
***T. verrucosum***	1	0,5%
**Total**	207	100

## Discussion

L´objectif de notre travail était d´étudier les caractéristiques épidémiologiques et mycologiques des TCC dans la région de Tunis. Nous avons constaté qu´elles touchent majoritairement les enfants d´âge prépubertaire < 12 ans (96%, n=201) avec une prédominance masculine. La forme clinique la plus fréquente était la teigne tondante à grandes plaques. Le diagnostic s´est basé sur l´examen direct et la culture. Nous avons noté la prédominance de l´espèce zoophile *M. canis*. L´identification moléculaire était concordante avec la macroscopie et la microscopie pour les 4 isolats. La prévalence des TCC était de 44,3% (n=210). L´étude de Makni *et al*. réalisée dans la région de sfax sur 13 ans, a rapporté une prévalence des TCC de 42% [3] et celle de Kallel *et al*. dans la région de Tunis sur 10 ans a rapporté une prévalence de 59,18% [4]. Des études maghrébines rapportent une prévalence proche de la nôtre de 33,48%, 43,85% et 62,4% pour respectivement Hamroune *et al*. (Algérie) [5], Oudaina *et al*. (Maroc) [6] et Bendjaballah *et al*. (Algérie) [7]. L´âge moyen de nos patients était 6,2 ans avec des extrêmes allant de 1 à 29 ans. Kallel *et al*. ont trouvé qu'un âge moyen proche (6,28 ans) [4]. Cette prédominance des bas âges est retrouvée également dans les pays du Maghreb [8], d´Afrique subsaharienne [9, 10] et de l´Europe [11]. Elle est due aux propriétés fongistatiques des triglycérides, des chaînes des acides gras, du sébum et des hormones sexuelles. Ce qui explique la guérison spontanée des malades à la puberté [12].

La prédominance masculine notée dans notre série était rapportée dans la plupart des études tunisiennes [4, 12], magrébines [6, 7], africaines [9], européennes [11] et américaines [13]. Ceci peut être expliqué par une chevelure plus courte des garçons (contamination plus facile par les spores) et par le contact plus important par rapport aux filles avec les animaux domestiques ou errants qui sont souvent des porteurs asymptomatiques [14]. Par ailleurs, l´atteinte féminine retrouvée dans quelques études serait due à l´échange de foulards, de bonnets ou d´outils de coiffure [12]. La teigne tondante à grandes plaques était l´aspect clinique le plus fréquemment observé (88%, n=184), de même pour Kallel *et al*. (65,9%) [4]. Dans les régions de Sousse et de Sfax, l´étude de Saghrouni *et al*. et celle de Makni *et al*. ont noté la prédominance des teignes tondantes trichophytiques dans 66,5% et 68,3% respectivement [3, 12]. L´examen direct était positif dans 87% des cas (n=182) dans notre étude et dans 93,95% des cas dans l´étude de Kallel *et al*. [4]. Par contre, la sensibilité de l´ED était plus basse dans l´étude de Saghrouni *et al*. (47,9%) [12]. En cas de positivité, l´ED permet de poser un diagnostic immédiat. Ce qui permet au clinicien de démarrer le traitement approprié sans attendre les résultats des cultures et limiter ainsi le risque de contamination de l´entourage [15].

Dans notre série, La sensibilité de la culture était de 98 % (n=205). Pour Kammoun *et al*., et Kallel *et al*., la sensibilité de la culture était de 90% et 96% respectivement [4, 16]. Par contre, elle était plus faible dans l´étude de Saghrouni (53,2%) [12]. La culture a permis de rattraper le diagnostic de TCC dans 28 cas dans notre étude. Elle représente est un complément indispensable de l´ED puisque la prophylaxie et le traitement varient en fonction de l´espèce isolée [15]. Cependant, dans notre série, la culture a été négative dans 3 cas avec un ED positif, cela pourrait être expliqué par des problèmes techniques comme l´ensemencement d´un matériel peu parasité ou la contamination de la culture par des moisissures à pousse rapide inhibant celle des dermatophytes beaucoup plus lente. Dans notre série, la culture a permis l´isolement et l´identification de 5 espèces de dermatophytes. *M. canis* était l´espèce dominante (87%, n=182), comme pour l´étude de Kallel *et al*. (67%) [4]. *T. violaceum* était prédominante dans les études maghrébiens dans 78% et 66,1% des cas selon Oudaina *et al*. et Bendjaballah *et al*. respectivement [6, 7]. L´étude belge de Vujovic *et al*. a rapporté la prédominance des teignes microsporiques à *M. audouinii* dans 60,8% des cas [17]. Le tableau 4 résume la répartition selon les espèces isolées dans les TCC dans notre étude comparée à la littérature. Le spectre des dermatophytes responsables des TCC n´a cessé de se modifier au fil des années. Ce qui souligne l´importance des études épidémiologiques étalées plusieurs années comme la nôtre. Notre étude a permis de consolider les données locales sur les TCC dans la région de Tunis. En plus, elle montre l´importance de l´introduction de la biologie moléculaire pour le diagnostic de ces mycoses ce qui permettrait d´accélérer la prise en charge des malades. Cependant, étant rétrospective, cette étude s´est limitée aux données contenues dans les registres dont certains ont été incomplets. Il aurait été mieux encore de faire une étude longitudinale et prospective des TCC.

**Tableau 4:** la répartition selon les espèces isolées dans les teignes du cuir chevelu dans notre étude comparée à la littérature

Etude Année (Pays) Espèce	Notre	Kallel 2017 (Tunisie)	Dridi 2015 (Tunisie)	Makni 2008 (Tunisie)	Saghrouni 2011 (Tunisie)	Oudaina 2011 (Maroc)	Ben jaballah 2014 (Algérie)	Nidiaye 2015 (Sénégal)	Vujovic 2014 (Belgique)
***M. canis***	**89%**	**67%**	**52,27%**	29,2%	29,3%	13,5%	32,33%	6,36%	-
***M. gypseum***	0,5%	0,22%		0,04	0,1%	6/1299	-	-	-
***T. violaceum***	9%	31,68%	18,18%	**68%**	**66,7%**	**78%**	**66,1%**	-	**-**
***T. mentagrophytes***	1,5%	0,66%	6,81%	1,34%	1,1%	1,8%	1,50%	4,6%	11,2%
***T. verrucosum***	0,5%	-	6,81%	0,7%	0,6%	-	-	-	-
***T. soudanense***	-	-		1,23%		(2/1299)	-	**56,18%**	-
***M .audouinii***	-	0,22%	4,54%	0,1%	0,03%	(2/1299)	-	-	**60,8%**
***T. rubrum***	-	-	2,27%	0,2%	0,2%	3%	-	18,37%	-
***T. schoenleinii***	-	0,22%	-	0,1%	1,6%	2 ,3%	-	-	-
***T. equinum***	-	-	-	-	-	(1/1299)	-	-	-
***T. tonsurans***	-	-	-	0,3	0,2%	(2/1299)	-	-	11,2%
***M. soudanense***	-	-	-	-	-	(2/1299)	-	-	20,8%
***T. quinckeanum***	-	-	-	0,02%	-	-	-	-	-
***M. nanum***	-	-	-	-	0,01%	-	-	-	-
***T. erinace***	-	-	1,13%	-	-	-	-	-	-

## Conclusion

Les teignes du cuir chevelu représentent l´infection fongique la plus rencontrée chez l´enfant avant la puberté. Notre étude montre l´émergence des dermatophytes zoophiles particulièrement *M. canis*, qui deviennent de plus en plus fréquents au profit des espèces anthropophiles. L´examen mycologique est essentiel pour la confirmation diagnostique, la surveillance épidémiologique des dermatophytes selon les régions et la prise en charge thérapeutique.

### Etat des connaissances sur le sujet

- Les teignes du cuir chevelu (TCC) constituent la principale mycose superficielle de l´enfant avant la puberté;
- Les TCC posent un problème sérieux de santé publique dans les pays en voie de développement;
- La distribution des dermatophytes en cause change avec le temps et la région.

### Contribution de notre étude à la connaissance

- Notre étude rapporte une caractérisation actualisée épidémiologique et mycologique des TCC dans la région de Tunis;
- Notre étude montre l´émergence des dermatophytes zoophiles particulièrement Microsporum canis dans la région de Tunis;
- L´examen mycologique est essentiel pour la confirmation du diagnostic, la surveillance épidémiologique des dermatophytes selon les régions et la prise en charge thérapeutique.
